# Thermal Failure Analysis of Fiber-Reinforced Silica Aerogels under Liquid Nitrogen Thermal Shock

**DOI:** 10.3390/molecules23071522

**Published:** 2018-06-24

**Authors:** Ai Du, Mingfang Liu, Shangming Huang, Conghang Li, Bin Zhou

**Affiliations:** 1 Shanghai Key Laboratory of Special Artificial Microstructure Materials and Technology, Tongji University, Shanghai 200092, China; liumfsmile@126.com; 2 School of Physics Science and Engineering, Tongji University, Shanghai 200092, China; 3 Key Laboratory of Graphene Technologies and Applications of Zhejiang Province, Ningbo Institute of Materials Technology & Engineering (NIMTE), Chinese Academy of Science, Ningbo 315201, China; 11Shangming@sina.com; 4 Laboratory of Space Mechanical and Thermal Integrative Technology, Shanghai Institute of Satellite Engineering, Shanghai 200240, China; lch2414@126.com

**Keywords:** thermal shock, fiber-reinforced aerogel, hydrophilic, hydrophobic

## Abstract

Aerogel materials are recognized as promising candidates for the thermal insulator and have achieved great successes for the aerospace applications. However, the harsh environment on the exoplanet, especially for the tremendous temperature difference, tends to affect the tenuous skeleton and performances of the aerogels. In this paper, an evaluation method was proposed to simulate the environment of exoplanet and study the influence on the fiber-reinforced silica aerogels with different supercritical point drying (SPD) technology. Thermal conductivity, mechanical property and the microstructure were characterized for understanding the thermal failure mechanism. It was found that structure and thermal property were significantly influenced by the adsorbed water in the aerogels under the thermal shocks. The thermal conductivity of CO_2_-SPD aerogel increased 35.5% after the first shock and kept in a high value, while that of the ethanol-SPD aerogel increased only 19.5% and kept in a relatively low value. Pore size distribution results showed that after the first shock the peak pore size of the CO_2_-SPD aerogel increased from 18 nm to 25 nm due to the shrinkage of the skeleton, while the peak pore size of the ethanol-SPD aerogel kept at ~9 nm probably induced by the spring-back effect. An 80 °C treatment under vacuum was demonstrated to be an effective way for retaining the good performance of ethanol-SPD aerogels under the thermal shock. The thermal conductivity increases of the ethanol-SPD aerogels after 5 shocks decreased from ~30 to ~0% via vacuum drying, while the increase of the CO_2_-SPD aerogels via the same treatments remains ~28%. The high-strain hardening and low-strain soften behaviors further demonstrated the skeleton shrinkage of the CO_2_-SPD aerogel.

## 1. Introduction

Due to the hierarchical, nanoporous microstructure and many unique properties, aerogel is different from ordinary materials and is even considered to be a new state of matter [1]. These unique materials exhibit many fascinating properties, which include very low thermal conductivity [2,3,4,5,6,7,8]. Since it was first prepared by Kislter in 1931 [9], silica aerogel had been applied in various fields especially in aerospace engineering as high performance thermal insulation materials [10,11], because it has high endurance in harsh environments such as high temperature, low temperature or alternating temperature, etc. [12,13] Silica aerogel was first used on the Mars Rover, Sojourner as part of the Pathfinder mission in 1997 [12,14].

Especially for Mars exploration, the landing site for the exoplanet has a surrounding temperature ranging from −84 °C to 27 °C. What is worse, this temperature may turn as low as to −100 °C. [11,15,16] The great temperature variation is a great challenge for the thermal insulation materials. The environment conditions must be considered in the design of materials to retain their heat resistance performance after the thermal shock from the dramatically varied temperature.

However, the thermal insulation application is greatly restricted by silica aerogel’s brittleness. Strategies including chemical grafting [17], functionalization [18] and fiber-reinforcement [19] are usually used to improve their performance. As a promising material, fiber-reinforced silica aerogel has been used for the high performance thermal insulation [20], which is employed in container for liquid rocket propellants protecting the liquid fuel of space shuttle, ensuring of the spacecraft work normally after entering the high temperature and low temperature environment of all parts [21]. NASA has conducted many astronautic missions by using this type aerogel as the thermal insulator [12]. X Yang. et al. mainly focused on the relationship between compression properties and the rising temperature (25~800 °C), and the possible mechanism for the stress relaxation of fiber-reinforced aerogel composite [9]. However, little attention has been paid to the influence of thermal shock processes on the microstructure and mechanical performances of aerogels in the past.

In this paper, we studied the thermal failure analysis of the fiber-reinforced silica aerogels under liquid nitrogen thermal shock simulating the environment of exoplanet. Two kinds of aerogels prepared by with different supercritical point drying (SPD) technology were compared to study the different failure behaviors. Meanwhile, to avoid the influence of the liquid on the microstructure, the aerogels were covered with Zip-lock bags before being immersed into the liquid nitrogen. Thermal conductivity, the microstructure and mechanical property were evaluated before and after the thermal shock processes.

## 2. Results and discussion

### 2.1. Supercritical Point Drying (SPD) and Hydrophobicity

**Figure 1:**
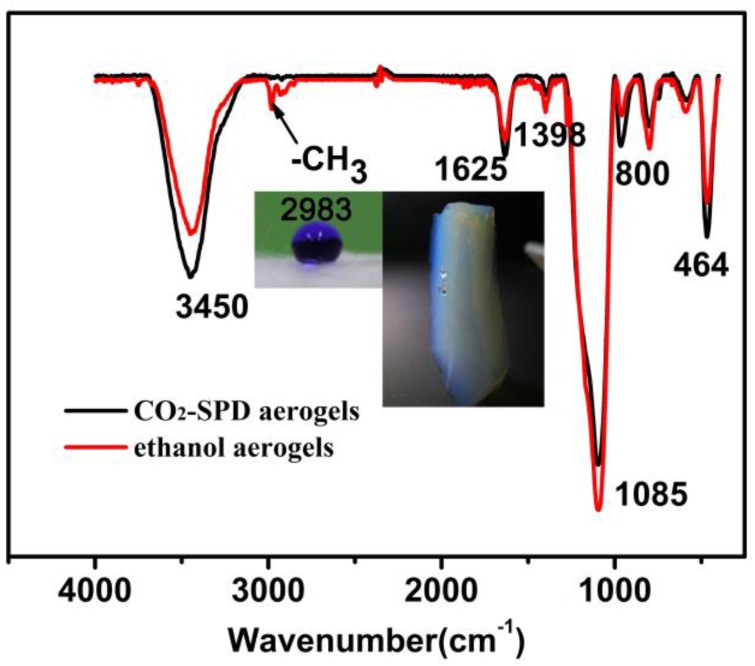
FT-IR spectra of the CO_2_-SPD and ethanol-SPD aerogels. Insets are the photographs of a water drop on the surface of the ethanol-SPD aerogels, the contact angle is about 140° and the water drop could stick to the surface.

Fourier transform infrared spectroscope (FTIR) and contact angle measurements can provide solid proof of group on the sample surface. It is evident from Figure 1, the strong peak near 1085 cm^−1^, the weak peak near 800 cm^−1^ and 464 cm^−1^ are assigned to the asymmetric and symmetric bending vibration of the Si–O–Si bonds [22,23]. The absorption peaks near 2983 cm^−1^ and 2900 cm^−1^ are due to the terminal –CH_3_ groups. In addition, since there were a lot of –CH_3_ group on the sample surface, it will perform hydrophobic characteristics in ethanol-SPD aerogel [6], while there is no peak near 2983 cm^−1^ and 2900 cm^−1^ in CO_2_-SPD aerogel [24]. It could be seen that the ratio of peak intensity (RPI) of Si–OH groups (965 cm^−1^) and Si–O–Si groups (1085, 800, 558 cm^−1^) differs [25]. The RPI of the ethanol-SPD aerogel sample is smaller than the RPI of the CO_2_-SPD aerogel sample, indicating partial conversion from Si–OH to Si–O–Si groups on the surface. However, this hydrophobicity of the ethanol-SPD aerogels may mainly attribute to the microstructure. There should be strong a interaction between the skeletons and water which is the driving force to adsorb the water. As shown in the inset of Figure 1, the water drop can adhere to the surface of ethanol-SPD silica aerogel, but there is no obvious infiltration into the surface.

Figure 1 also reveals the photographs of water droplets placed on the ethanol-SPD aerogel, and the corresponding contact angle with water is 140°. It is worth noting that the contact angle of the CO_2_-SPD aerogel could not be measured since it shrinks seriously into the white powder when it contacts the water drop. It is notable that the wide band near 3450 cm^−1^ and the peak at 1625 cm^−1^ were assigned to the stretching vibration of O–H group and bending vibration of H–O–H [24,26,27]. That indicates that both the CO_2_-SPD and ethanol–SPD aerogels contain adsorbed water.

### 2.2. Thermal Conductivity Failure Process

**Figure 2:**
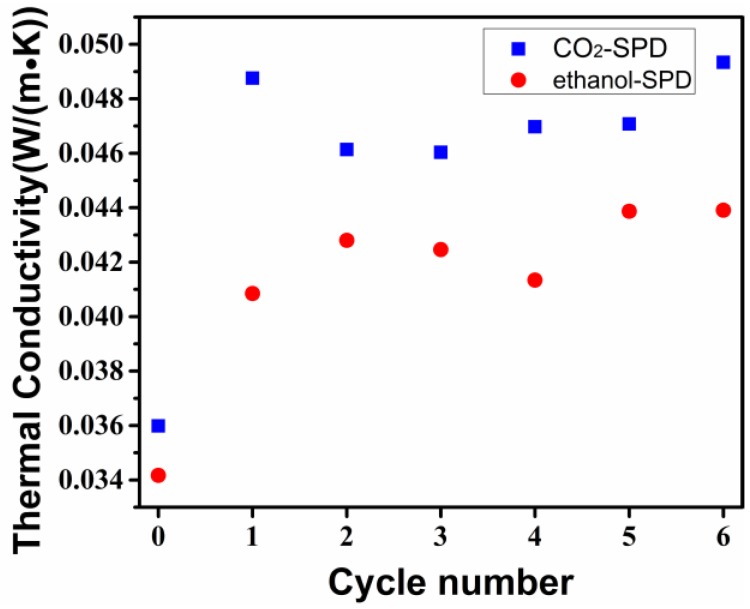
Thermal conductivity evolution of the CO_2_-SPD and ethanol-SPD aerogels via thermal shocks.

As described in Figure 2, it is the thermal conductivity of the CO_2_-SPD and ethanol-SPD aerogels under liquid nitrogen thermal shocks in the air. It indicates that thermal conductivity of the CO_2_-SPD and ethanol-SPD aerogels increase sharply after the first cycle of liquid nitrogen thermal shock. The thermal conductivity of the CO_2_-SPD aerogel cycle increases from 0.036 W/(m·K) to 0.049 W/(m·K), while the ethanol-SPD aerogel increases from 0.034 W/(m·K) to 0.041 W/(m·K). After the next several cycles, the average values of the CO_2_-SPD and ethanol-SPD aerogels are ~0.048 W/(m·K) and ~0.043 W/(m·K), respectively.

It is found that the thermal conductivity of CO_2_-SPD aerogel increases by 36% after the first shock and keep in a high value, while that of the ethanol-SPD aerogel increase by 20% and remain in a relatively low value after multiple shocks (Figure 2). For the aerogel composite, the nanoporous structure greatly extends the heat transfer path and forms the infinite heat conduction path, which results in a very low solid thermal conductivity [20,28]. Furthermore, with the cycles increased, the thermal conductivity of samples is increased. The result indicates that the microstructure of both samples may have changed under the liquid nitrogen shock.

### 2.3. Microstructure Evolution during the Shock Processes

**Figure 3:**
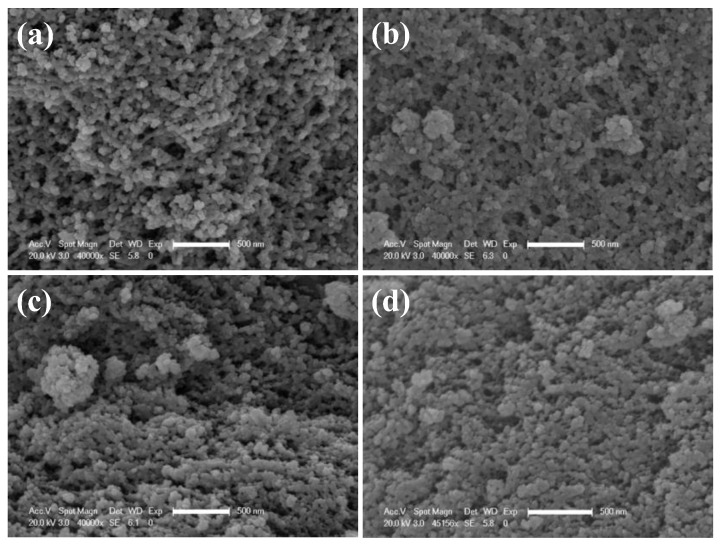
SEM images of CO_2_-SPD aerogel before (**a**) and after (**b**) the first thermal shock and ethanol-SPD aerogels before (**c**) and after (**d**) the first thermal shock (Scale bar: 500 nm).

The microstructure of CO_2_-SPD and ethanol-SPD aerogels before and after thermal shock are provided in Figure 3. The CO_2_-SPD aerogels before shock has highly porous networks and no obvious compact structure, as shown Figure 3a. After the first thermal shock, the networks of CO_2_-SPD aerogels tend to shrink. The pore size becomes larger and the skeletons becomes thicker (Figure 3b). Figure 3c,d are the scanning electron microscopy (Brinker, C.J.) images of ethanol-SPD aerogels before and after the first thermal shock. It seems that skeleton and pore size have no obvious change.

**Figure 4:**
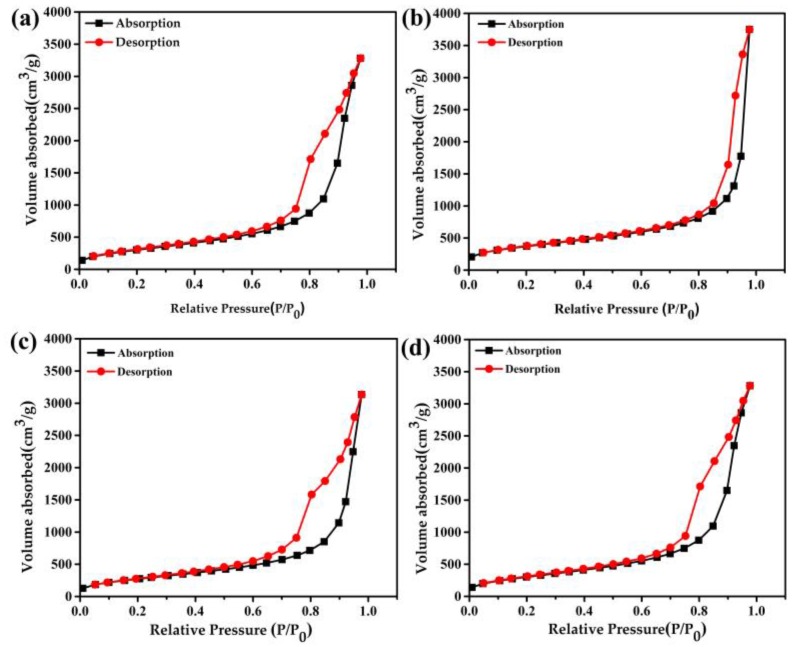
Nitrogen adsorption and desorption isotherms of CO_2_-SPD aerogels before (**a**) and after (**b**) the first thermal shock, and ethanol-SPD aerogels before(**c**) and after (**d**) the first thermal shock.

The specific surface areas and pore size distribution of the two kinds of aerogel are studied. The adsorption and desorption branches of the isotherms is analyzed by applying the Brunner-Emmet-Teller (BET) and Barrett-Joyner-Halenda (BJH) methods [29]. The relevant results of the experiment are listed in Table 1. The nitrogen adsorption-desorption isotherms curves were provided in Figure 4.

**Figure 5:**
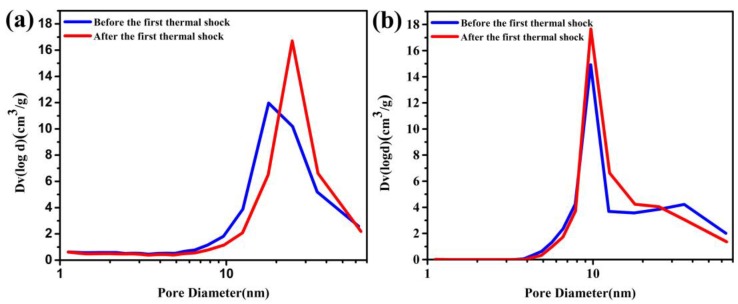
(**a**) Pore size distribution of the CO_2_-SPD aerogels before and after the first thermal shock; (**b**) The ethanol-SPD aerogels before and after the first thermal shock.

The peak pore size of the CO_2_-SPD aerogels increases from 18.0 nm to 24.9 nm after the first shock, while the peak pore size of the ethanol-SPD aerogels remains about 9.7 nm (Figure 5 and Table 1). It indicates that the skeleton of the CO_2_-SPD aerogels becomes thicker and its pore size turn into bigger, while the structure of the ethanol-SPD aerogels has no obvious change. Moreover, specific surface area of the CO_2_-SPD aerogels reduces from 1521 m^2^/g to 1316 m^2^/g (Sample a and c), while specific surface area of the ethanol-SPD aerogels increase from 1002 m^2^/g to 1117 m^2^/g (Sample b and d).

Compared with the sample before thermal shock, the skeleton becomes thicker and pore size turns into larger for the CO_2_-SPD aerogel (Figure 3a,b). While skeleton and pore size of the ethanol-SPD aerogel has barely changed (Figure 3c,d). Specifically, the specific surface areas reduce by ~200 m^2^/g. In addition, peak pore size of the CO_2_-SPD aerogel increases from ~18 nm to ~25 nm (Sample a and b in Table 1) after thermal shock. Pore size of the CO_2_-SPD aerogel becomes larger and the number of pores decrease in the same volume [30]. It suggests that parts of pores are collapsed, which increase the surrounding space. Meanwhile, decrease in specific surface areas may also attribute to destructor of the microstructure after thermal shock conformed to the Figure 3a,b. For ethanol-SPD aerogel, the specific surface area increases by 100 m^2^/g, which may be induced by the removal of adsorbed water in the frozen process. In addition, the peak pore size of the ethanol-SPD aerogel kept at ~9 nm. They indicate that part of pore size of ethanol-SPD aerogel diminished and part of the big pores reduce to the mesoporous after thermal shock. (Sample c and d in Table 1). In brief, the CO_2_-SPD aerogel suffers more serious from failure process than ethanol-SPD aerogel.

In the process of studying the failure mechanism, the result that hydrophilic and hydrophobic aerogels contain a small amount of water can be found by the FT-IR spectra (Figure 1). During the thermal shock process in liquid nitrogen, there may be driving force derived from the deformation of adsorbed water (including ice state) during the temperature changes. We infer that the volume expansion and the certain damage of frame are caused by freezing water.

### 2.4. Supposed Failure Mechanism of Aerogels during the Shock Processes

For further demonstrating the thermal failure of the CO_2_-SPD samples, with the condition of high strain and low strain before and after the first thermal shock. The mode that aerogels are elastically deformable by more than 15% in an almost reversible manner, we defined it as ‘the condition of high strain’. The samples were characterized by universal materials testing machine. The mode that aerogels are elastically deformable by less than 5% in an almost reversible manner, we defined it as ’the condition of low strain’. Dynamic thermomechanical analysis (DMA) was used to study the mechanical behaviors.

**Figure 6:**
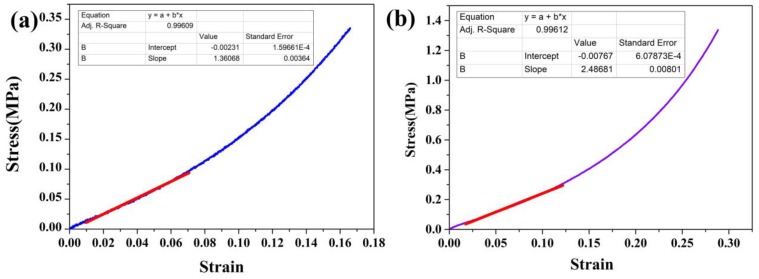
Stress-stain curve of the CO_2_-SPD aerogels before (**a**) and after (**b**) the first thermal shock.

Stress-strain curves of the CO_2_-SPD aerogel are exhibited in Figure 6a,b. The samples before and after thermal shock are compressed up to 16% and 28% of its original height, respectively. The porous structure can be deformed elastically at the beginning of the compression test and the slope in this range is used to calculate Young’ modulus [31]. As shown in tables of Figure 6, for the CO_2_-SPD aerogel, the slope of the sample after thermal shock is 2.49 MPa while the slope of the sample before thermal shock is 1.36 MPa.

The young’s modulus of the CO_2_-SPD aerogel is measured by adopting the compression mode of universal material tester. The large range of deformation is primarily attributed to the mesoporous silica aerogel. It is recognized that the ultra-low density nanoporous silica micro area deformation can be measured by uniaxial compression mode [32]. The increase in modulus may attributed be the shrinkage of aerogel grain, which agrees with the microstructure results.

**Figure 7:**
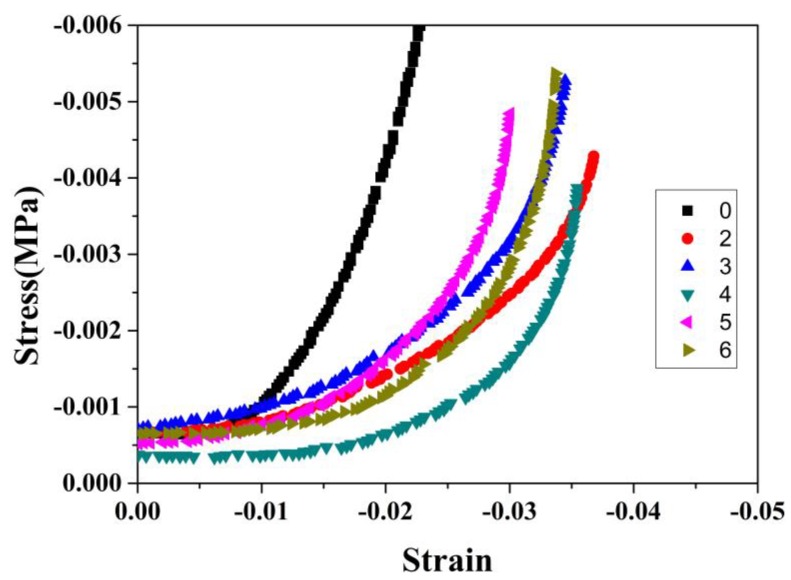
Low strain recovery testing results of the CO_2_-SPD aerogel along with the cycles of thermal shock.

Figure 7 shows continuous load-displacement curves for CO_2_-SPD aerogel with different cycles of thermal shock tests at room temperature and cryogenic temperature under liquid nitrogen. From Figure 7, when the strain is −0.02, the corresponding stress of the sample is 0.0041 MPa without thermal shock and the slope of the stress strain curve is 0.444. While the corresponding stress −0.02 of sample after the next several cycles is almost lower than 0.0016 MPa and the slope of the stress strain curve is lower than 0.217. Overall, the slope of the stress strain curves exhibit irregular with the cycles of shock, which may be caused by dropped aerogel powder in surface. That means that in the low-strain modulus of the aerogel decreases but the high-strain modulus increases obviously.

**Figure 8:**
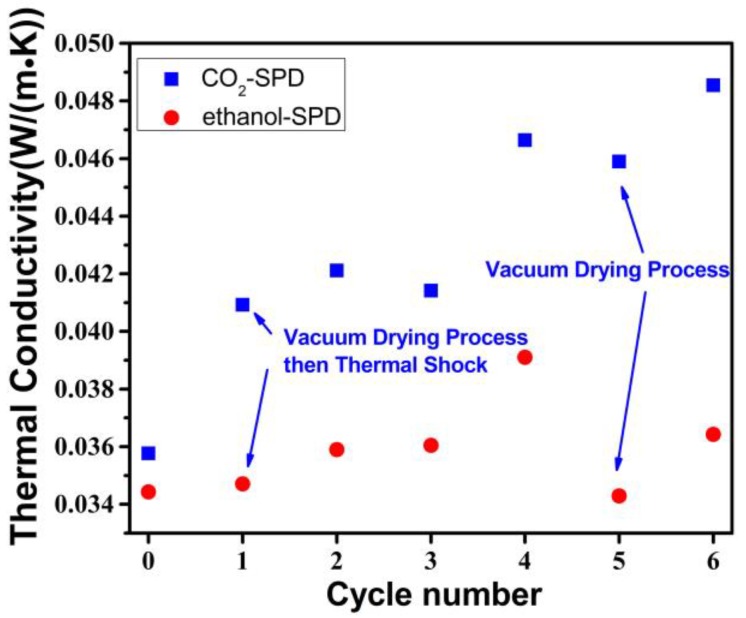
Thermal conductivity changing with cycle times with vacuum drying process.

To further demonstrate the mechanism, the aerogel was treated at 80 °C under vacuum environment before conducting shock experiments to avoid the effect of the free water. The reason why the higher temperature was not chosen to eliminate the adsorbed water is to avoid the possible thermal failure induced by the high temperature. It is observed that the thermal conductivity of the CO_2_-SPD aerogel increases from 0.036W/(m∙K) to 0.041 W/(m∙K) in Figure 8, while ethanol-SPD aerogel is increasing from 0.034 W/(m∙K) to 0.035 W/(m∙K) after the first shock. Compared to the initial state value, the thermal conductivity of two samples have increased greatly after four cycles, which were 0.047 W/(m∙K) and 0.039 W/(m∙K), respectively.

The aerogel was treated at 80 °C under vacuum environment again (5th Cycle in Figure 8). The thermal conductivity of the CO_2_-SPD aerogel has a slight decrease from 0.047 W/(m∙K) to 0.046 W/(m∙K), while the thermal conductivity of the ethanol-SPD aerogel decreases from 0.039 W/(m∙K) to 0.034 W/(m∙K). This indicates the free water in the aerogels may greatly influence the failure behaviors of both CO_2_-SPD and ethanol-SPD aerogels after thermal shocks. After vacuum drying treatment, the ethanol-SPD aerogel exhibit recovered thermal failure behavior, while the CO_2_-SPD aerogel shows an irreversible behavior that the thermal conductivity is kept in a high value. This indicates it may be the key that the adsorbing water to the driving force of the failure.

The water content may significant affect the thermal failure behavior. However at present, we have no way to adjust the environment humidity during the characterization of thermal conductivity. Thus further studies on the influence of the water content on the failure behavior could not be carried out in this work.

**Figure 9:**
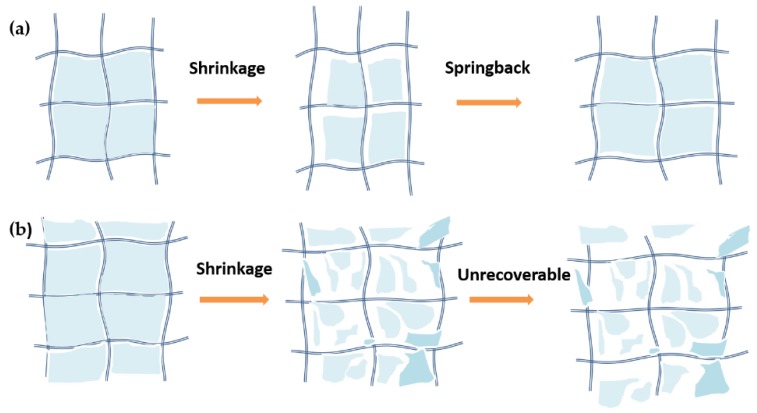
Schematic diagram about recovered and irreversible process of the ethanol-SPD (**a**) and CO_2_-SPD (**b**) fiber-reinforced silica aerogel.

The ethanol-SPD aerogel suffers less damage because of the unique “Spring-back” effect [33] (Figure 9a), which is confirmed by SEM images and results of nitrogen adsorption and desorption isotherms. The spring-back effect (defined by Brinker [34] in 1990) is a well-known concept explaining the phenomena of the shrinkage and spring-back of the microstructure of the aerogels. Thus this is different from the pressing and recovery of the elastic sample during the load-unload measurement. It is notable that only the microscopic spring-back behavior of the aerogel grains inside the composite samples is discussed in this paper, but not the whole samples. There is obvious shrinkage of frame at first and weak adhesion between skeleton in aerogel during the freezing process under liquid nitrogen. Then, the skeleton will be elastically separated. The ethanol-SPD aerogel exhibits the spring-back effect with aerogel grains after 80 °C vacuum drying treatment (6th in Figure 8), and the treatment make the thermal conductivity decrease as low as the untreated samples. By removing the adsorbed water, the thermal conductivity increases of the ethanol-SPD aerogel after 5th shocks decrease from ~30 to ~0%. On the contrary, the CO_2_-SPD aerogel grains do shrink during the thermal shock but cannot spring back to its original state, which becomes unrecoverable. Therefore, the macroscopic mechanical properties and the mechanical properties of low strain situation are carried on for CO_2_-SPD aerogel before and after thermal shock. The increase of the CO_2_-SPD aerogel via the same treatments remains ~28%.

The high-strain hardening and low-strain soften behaviors the skeleton shrinkage of the CO_2_-SPD aerogel is further demonstrated by the high-strain hardening and low-strain soften behaviors. On the one hand, as provided in Figure 5, for CO_2_-SPD aerogel, the Young’ modulus of sample after thermal shock (2.49 MPa) is 83.09% higher than the slope of sample before thermal shock (1.36 MPa), which means that thermal shock obviously contribute much to the increase in stiffness. The shrinkage increases the density of the aerogel, which lead to a large modulus. Also, the thicker skeleton and the larger pore size may make the aerogel rigid when conducting the high-strain compression. On the other hand, the slope of the first curve is obvious higher than the other cycles under the low strain (weak stability) in Figure 6. During the cycles of shock, the Young’s modulus decreases and loses the regularity. It indicates that silica aerogel grains do shrink, while fiber mats remain unchanged because of its stiffness. Some aerogel near the surface will be fractured by shrinkage of skeleton or external force. The fragment may separate from the fibers of mats after thermal shock under the low strain (Figure 9b). Therefore, when conducting low-strain compression, just surface fiber mats is pressed. Lack of the support of the aerogel, the modulus of the samples will decrease naturally.

To further demonstrating the mechanism, the aerogels were treated at 80 °C under vacuum environment before conducting shock experiments. It indicates that after the first shock, the thermal conductivity of the CO_2_-SPD and ethanol-SPD aerogels is increased only 14.0% and 0.4% (Figure 8), and the advantages are obvious in comparison with the sample before thermal shock in air (Figure 2). After 5 cycles of thermal shock, the thermal conductivity of the CO_2_-SPD had little change under the vacuum drying, while that of ethanol-SPD aerogel can restored nearly to its original state. It is revealed that the processes of the CO_2_-SPD grains are irreversible during the temperature changes. While microstructure and thermal conductivity of the ethanol aerogel grains could be recovered due to the spring-back effect after vacuum drying. (Figure 9a,b) An 80 °C treatment under vacuum was demonstrated be an effective way for retaining the high stability during the thermal shock between high temperature differences.

Accordingly, for the space exploration, it is necessary to use the aerogel with hydrophobic treatment. Despite the water content in the exoplanet environment (such as the Mars) is very few, the adsorbing water cannot be avoided during the storage in the ground. Therefore, when planning to use the aerogel, it is necessary to conduct vacuum drying treatment to reduce the damage or the failure during the shock processes.

It is worth noting that the water content may be an important parameter affecting the thermal failure behavior of the aerogels. However, in this work, the adsorbed water content is very low, which could not measure its precise amount via normal weighting method or even FTIR spectra. Thus, work about the influence of water content on the failure process could be carried out to further clear the mechanism.

## 3. Material and Methds

### 3.1. Chemicals

Tetraethoxysilane (TEOS, AR), ethanol (AR), hydrochloride (HCl, 36~38%) and NH_4_OH (ammonia solution, 25~28% NH_3_) were purchased from Sinopharm Chemical Reagent Co. Ltd., Shanghai, China. Deionized water (H_2_O) was purchased from School of Environmental Science and Engineering, Tongji University. The glass fiber mats (~100 mg·cm^−3^) were provided by Tianjin Morgan-Kundom Hi-Tech Development Co. Ltd., Tianjin, China. All the chemical reagents were used as received.

### 3.2. Synthesis of Hydrophilic and Hydrophobic Fiber-Reinforced Silica Aerogels

By the two-step acid-base sol-gel reaction with the glass fiber mats, combined with supercritical point drying technology, [6] we prepared the CO_2_-SPD and ethanol-SPD fiber-reinforced silica aerogels.

SiO_2_ sol was used by base/acid two-step catalyzed process similar to the previous works [9,35,36]. In the first step, TEOS, ethanol, H_2_O, and HCl were mixed in a molar ratio of 1:2.4:1.5 × 10^−5^. The TEOS was hydrolyzed in a solution under reflux for 20 h at 95 °C. After refluxing, mixed solution was distilled off for 4 h at 105 °C and the CS (condensed silica precursor) oil collected. The residual CS contains about 9.35 × 10^−3^ mol silicon per milliliter of CS by calculation. In the second step, silicon of CS, ethanol, H_2_O, and NH_4_OH were mixed in a molar ratio of 1:27.5:8.9:2.1 × 10^−2^. The sol was poured into glass fiber mats in LOCK&FRESH (200 mm × 200 mm × 60 mm). In 1-h, the frameworks of the composite were gradually formed. The mixture of SiO_2_ sol completed gelation with glass fiber mats. The mixture was aged in 30 °C for one day and washed in ethanol for another day.

On the one hand, alcogels with the glass fiber mats were supercritically dried using CO_2_ as drying solvent. The CO_2_-SPD fiber-reinforced silica aerogels were prepared at 40 °C and 10 MPa. On the other hand, supercritical point drying was conducted with ethanol at 255 °C and 9 MPa to eventually get the ethanol-SPD fiber-reinforced silica aerogels. Density of the CO_2_-SPD and ethanol-SPD fiber-reinforced silica aerogels are about 140 mg·cm^−3^ and 150 mg·cm^−3^, respectively. The bulk density of the samples was calculated from weight and volume of the samples.

### 3.3. The Thermal Shock Experiments

During the thermal shock experiments, it was ensured that samples and temperature probe were not exposed to the liquid nitrogen. At first, CO_2_-SPD and ethanol-SPD fiber-reinforced silica aerogels (Hereinafter referred to as “the CO_2_-SPD and ethanol-SPD aerogels”, respectively) were sealed in Zip-lock bags. Company with probe, they were put in a bigger one. Then, they were submerged under liquid nitrogen and started timing after the temperature instrument displayed −196 °C for 10 min. After 1 h of low temperature shock, they were removed at room temperature (25 °C). After they recovered at room temperature and were kept half an hour, samples started to be tested. The above process called a cycle. To simulate planetary surface environment, we took several cycles.

### 3.4. Characterization

Organic groups were investigated by a Fourier transform infrared spectroscope (FTIR, TENSOR27, Bruker, Karlsruhe, Germany) and spectra are recorded in the range of 4000–450 cm^−1^. The KBr squash technique was used to prepare each FTIR sample. Different sizes of samples for measurement were cut from the same sample. Thermal conductivity was measured on TPS 2500S thermal constants analyzer (Hot Disk, Uppsala, Sweden) at room temperature by several times measurements. The measuring precision is 0.001 W/(m·K). The size of samples was 15 mm × 15 mm × 8 mm. The morphology of the aerogels was observed by scanning electron microscopy (SEM, Philips-XL30FEG, Eindhoven, The Netherlands). Samples were fractured at room temperature and sputter coated with gold before the SEM observation. With fibers of the sample removed, the specific surface area and pore size distribution of the aerogels were obtained from nitrogen adsorption–desorption isotherms at 77 K, analyzed by using a Quantachrome Autosorb-1 analyzer (Quantachrome Instruments, Miami, FL, USA). All samples were outgassed by heating at 80 °C for 8 h under a vacuum before collecting adsorption and desorption isotherm data by using nitrogen as the adsorption at 77 K. The surface area of the sample was calculated by the Brunauer-Emmett-Teller (BET) method, and pore size distribution for the micro-pores and meso-pores was determined by modeling using the BJH theory. Low strain recovery testing was taken on a CMT 5105 universal materials testing machine. The elastic modulus was taken as the slope of the initial linear portion in the obtained stress–strain curve of the compression. The mechanical properties were studied by dynamic thermomechanical analysis (DMA 8000, PerkinElmer, Waltham, MA., USA). The size of samples for dynamic thermomechanical analysis is 9.0 mm × 8.0 mm × 4.5 mm. Low strain recovery testing was conducted according to the following steps: (1) Choosing compression testing mode and setting up the compression holder; (2) adjusting the balance distance between upper fixture and lower detector slightly shorter than the height of the samples; (3) descending the detector, placing the samples and rising the detector back to the balance position; (4) starting the test and recording the spring-back data. The detector will descend and record the negative stress and strain because of the spring-back compression mode. Contact angles (CA) were measured using a JC2000A optical contact-angle software.

## 4. Conclusions

As the attractive candidates for insulator, aerogels could be used for high-efficiency insulation under exoplanet environment. In this paper, liquid nitrogen-room temperature thermal shocks, which were used to simulate the environment of exoplanet, were carried repeatedly on fiber-reinforced silica aerogels. Effects of thermal shock on the properties and microstructures of the samples with different hydrophobicity were investigated. As expected, hydrophobic samples (ethanol-SPD aerogel) exhibit higher stability after the thermal shock. This could be explained that absorbed water is a key factor for the failure process in harsh environment. Moreover, the spring-back effects of methyl groups in ethanol-SPD silica aerogel aerogels make this process recoverable. At last, an 80 °C treatment under vacuum was demonstrated be an effective way for retaining the high stability during the thermal shock between high temperature differences.

## Figures and Tables

**Table 1:** The specific surface area and pore structure parameters of CO_2_-SPD aerogels before (**a**) and after (**b**) the first thermal shock, and ethanol-SPD aerogels before (**c**) and after (**d**) the first thermal shock. Here the aerogels used have been treated by removing the glass fibers.

Sample	Density (mg/cm^3^)	Specific Surface Area (m^2^/g)	Average Pore Size (nm)	Peak Pore Size (nm)	Pore Volume (cm^3^/g)
a	140	1521	16.3	18.0	6.1
b		1316	17.7	24.9	5.8
c	150	1002	19.4	9.7	4.9
d		1117	18.2	9.7	5.1
